# Red Cell Distribution Width to Red Blood Cell Ratio: A Novel Biomarker for Predicting 30‐Day Mortality in Type B Acute Aortic Dissection

**DOI:** 10.1155/emmi/5722036

**Published:** 2026-09-22

**Authors:** Jingjing Wang, Jun Zhou

**Affiliations:** ^1^ Department of Clinical Laboratory, Jiangsu Province (Suqian) Hospital, Suqian, Jiangsu, China; ^2^ Department of Laboratory Medicine, the First Affiliated Hospital of Nanjing Medical University, Nanjing, Jiangsu, China, njmu.edu.cn

**Keywords:** aortic dissection, mortality, prognosis, red blood cell count, red cell distribution width, Type B

## Abstract

**Background:**

Type B acute aortic dissection (BAAD) is associated with significant morbidity and mortality. Early identification of high‐risk patients remains challenging. This study aimed to investigate whether the ratio of red cell distribution width to red blood cell count (RRR) could serve as a novel prognostic biomarker for 30‐day mortality in patients with BAAD.

**Methods:**

This retrospective cohort study enrolled 126 BAAD patients between 2017 and 2019. The primary outcome was 30‐day mortality. To mitigate overfitting and identify robust predictors from a comprehensive panel of laboratory parameters, we employed the Boruta algorithm for feature selection, followed by variance inflation factor (VIF) analysis to assess multicollinearity. Variables with VIF > 5 were excluded. An optimal RRR cutoff was determined using receiver operating characteristic (ROC) analysis. The Boruta algorithm and multivariate logistic regression were used to identify independent predictors.

**Results:**

During the 30‐day follow‐up period, 16 deaths (12.7%) were recorded. Comparative analysis showed that nonsurvivors had significantly lower RBC counts and hemoglobin, higher RDW, and markedly elevated RRR compared to survivors (all *p* < 0.05). The AUC for RRR was 0.774 (95% CI: 0.672–0.876) (RBC AUC = 0.741; RDW AUC = 0.684). However, the prognostic performance of RRR was not statistically superior to that of RDW alone (DeLong’s test, *p* = 0.155) or RBC alone (DeLong’s test, *p* = 0.335). After Boruta selection and VIF‐based exclusion of collinear variables (AST and RBC, both VIF > 5), the final multivariable logistic regression model included RRR, ALT, PLT, and CREA. In multivariate analysis, only RRR remained independently associated with mortality within this model (OR = 3.059, 95% CI: 1.431–6.540, *p* = 0.004).

**Conclusion:**

The RRR, a simple index derived from routine blood tests, was independently associated with 30‐day mortality within our laboratory‐based model in patients with BAAD. Given this association, the RRR warrants prospective validation as a prognostic tool.

## 1. Introduction

Type B acute aortic dissection (BAAD) is a critical cardiovascular emergency characterized by dissection confined to the descending aorta, accounting for 25%–40% of all acute aortic dissection cases [1]. Despite significant advancements in diagnostic imaging and endovascular therapy, early mortality remains substantial, often exceeding 10% within 30 days [2, 3]. Prompt and accurate risk stratification is, therefore, essential to guide clinical management, particularly in distinguishing patients who require urgent intervention from those who may follow a more indolent course. Current risk prediction tools, which rely primarily on clinical and anatomical data, can be cumbersome and sometimes demonstrate limited accuracy in predicting early death.

Recent research has increasingly focused on inexpensive and readily available hematological and biochemical biomarkers to aid in diagnosis and prognosis. Red cell distribution width (RDW), a standard component of complete blood counts that quantifies erythrocyte size variation (anisocytosis), has demonstrated significant prognostic values in various cardiovascular conditions, including heart failure, infective endocarditis, and stroke [4, 5]. As a marker of impaired red blood cell homeostasis, RDW reflects underlying systemic inflammation, hypoxemia, and iron deficiency [6].

While RDW provides useful clinical information, its predictive capacity may be enhanced when combined with other red blood cell indices, such as the RDW‐to‐albumin ratio or the RDW‐to‐platelet ratio [7, 8]. The RDW‐to‐red blood cell count ratio (RRR) integrates two key physiological aspects: variability in erythrocyte size and the blood’s overall oxygen‐carrying capacity. An elevated RRR may indicate concurrent inflammatory dysregulation and relative anemia—a particularly detrimental state during an acute, high‐stress event such as aortic dissection, where tissue oxygenation is often compromised. Despite its plausible pathophysiological relevance, the prognostic utility of RRR in patients with BAAD has not been investigated.

Therefore, the objective of this study was to evaluate the association between the RRR and 30‐day mortality in patients diagnosed with BAAD.

## 2. Methods

### 2.1. Study Design and Population

This retrospective cohort study enrolled patients diagnosed with BAAD between 2017 and 2019 at the First Affiliated Hospital of Nanjing Medical University. The inclusion criteria were as sollows: (1) a diagnosis of BAAD confirmed by computed tomography angiography (CTA), (2) presentation in the acute phase (symptom onset within 14 days of admission), and (3) with complete blood count parameters. Exclusion criteria included Marfan syndrome, traumatic or iatrogenic dissection, intramural hematoma, a prior history of BAAD, severe heart failure, active malignancy, recent blood transfusion, and myocardial infarction. After screening, a final cohort of 126 patients was included for analysis.

### 2.2. Data Collection and Definitions

Baseline demographic and admission laboratory data were collected. All laboratory values were obtained from the first blood draw upon presentation to the emergency department, prior to any definitive intervention or surgery. Laboratory assessments comprised complete blood count parameters (white blood cell count [WBC], lymphocyte count [LY], monocyte count [MO], neutrophil count [NE], red blood cell count [RBC], hemoglobin [HGB], RDW, and platelet count [PLT]); liver and muscle enzymes (alanine aminotransferase [ALT], aspartate aminotransferase [AST], alkaline phosphatase [ALP], lactate dehydrogenase [LDH], and creatine kinase [CK]); metabolic markers (total bilirubin [TBIL], direct bilirubin [DBIL], total cholesterol [TC], triglycerides [TG], high‐density lipoprotein [HDL], low‐density lipoprotein [LDL], albumin [ALB]); renal function indices (urea nitrogen [UREA], serum creatinine [CREA]); and coagulation profiles (prothrombin time [PT], activated partial thromboplastin time [APTT], fibrinogen [FIB], thrombin time [TT], and D‐dimer). The platelet‐to‐creatinine ratio (PCR) was calculated as PLT divided by CREA. Mortality was verified through a combination of hospital death records, medical record review, and telephone interviews with patients or their families. Follow‐up was 100% complete for all 126 patients. While outcome assessors were not formally blinded to the RRR values given the retrospective nature of the study, the outcome data were collected from objective records, minimizing the risk of assessment bias. The RRR was defined as RDW (%) divided by RBC count (× 10^12^/L). Given the retrospective and anonymized nature of the study design, the Institutional Ethics Committee of the First Affiliated Hospital of Nanjing Medical University granted a waiver for informed consent (2021‐SR‐529). The waiver also covered the telephone follow‐up component, as patient contact was limited to outcome verification only. The investigation adhered to the ethical principles of the Declaration of Helsinki.

### 2.3. Statistical Analysis

Continuous variables are presented as mean ± standard deviation (normally distributed data) or median (interquartile range) (nonnormally distributed data), while categorical variables are expressed as frequencies (percentages). Normality was assessed using the Shapiro–Wilk test. Group comparisons were performed using Student’s *t*‐test or the Mann–Whitney U test for continuous variables and the chi‐square test for categorical variables, as appropriate. Receiver operating characteristic (ROC) curve analysis was used to determine the optimal RRR cutoff value for predicting 30‐day mortality. To mitigate overfitting and select stable predictive features from a comprehensive panel of laboratory parameters, we employed the Boruta algorithm (R package “Boruta”, Version 7.0.0; ntree = 500; maxRuns = 100; random seed = 42), a random forest‐based wrapper method that compares the importance of actual variables against randomly permuted “shadow” features. Following Boruta selection, we assessed multicollinearity among the selected variables using the variance inflation factor (VIF). Variables with VIF > 5 were considered to indicate severe collinearity and were excluded from the final model. The remaining variables were then entered into a multivariable logistic regression model to identify independent predictors of 30‐day mortality. A two‐sided *p* value < 0.05 was considered statistically significant. All analyses were conducted using SPSS software and R Version 4.0.3.

## 3. Results

### 3.1. Baseline Characteristics

A total of 126 patients with BAAD were enrolled, among whom 16 (12.7%) died within 30 days. Comparative analysis between survivors and nonsurvivors revealed that nonsurvivors had significantly lower RBC (4.01 vs. 4.48 × 10^12^/L; *p* = 0.002), lower HGB levels (125 vs. 138 g/L; *p* = 0.003), higher RDW values (13.35% vs. 12.70%; *p* = 0.018), and lower TC levels (3.83 vs. 4.42 mmol/L; *p* = 0.010). Most notably, the RRR was significantly elevated in nonsurvivors compared to survivors (3.51 vs. 2.84; *p* < 0.001) (Table 1). No significant differences were observed between the two groups regarding age, sex, WBC, PLT, or other laboratory parameters.

**TABLE 1:** Patient demographics and baseline characteristics.

Characteristic	Survivors (*N* = 110)	Nonsurvivors (*N* = 16)	*p* value
Gender (male), *n* (%)	88 (80.0%)	13 (81.3%)	0.999
Age (years), mean ± SD	55 ± 14	62 ± 15	0.132
WBC (× 10^9^/L), median (Q1, Q3)	9.9 (7.8, 12.6)	10.5 (7.8, 11.8)	0.747
LY (× 10^9^/L), median (Q1, Q3)	1.14 (0.86, 1.50)	0.99 (0.79, 1.42)	0.375
MO (× 10^9^/L), mean ± SD	0.80 ± 0.35	0.73 ± 0.32	0.400
NE (× 10^9^/L), median (Q1, Q3)	7.9 (5.8, 10.2)	8.3 (5.8, 10.0)	0.829
RBC (× 10^12^/L), median (Q1, Q3)	4.48 (4.02, 4.79)	4.01 (3.46, 4.30)	0.002
HGB (g/L), median (Q1, Q3)	138 (124, 147)	125 (105, 130)	0.003
RDW (%), median (Q1, Q3)	12.70 (12.27, 13.40)	13.35 (12.80, 14.00)	0.018
PLT (× 10^9^/L), median (Q1, Q3)	162 (139, 203)	154 (81, 185)	0.273
ALT (U/L), median (Q1, Q3)	18 (12, 32)	20 (15, 40)	0.636
AST (U/L), median (Q1, Q3)	20 (16, 27)	28 (18, 71)	0.071
ALP (U/L), median (Q1, Q3)	85 (69, 102)	84 (78, 98)	0.695
LDH (U/L), median (Q1, Q3)	234 (212, 279)	276 (220, 441)	0.093
CK (U/L), median (Q1, Q3)	68 (44, 122)	130 (58, 344)	0.066
TBIL (μmol/L), median (Q1, Q3)	15 (11, 21)	14 (11, 18)	0.881
DBIL (μmol/L), median (Q1, Q3)	5.30 (3.90, 8.10)	5.65 (4.50, 6.60)	0.736
TC (mmol/L), mean ± SD	4.42 ± 1.03	3.83 ± 0.75	0.010
TG (mmol/L), median (Q1, Q3)	1.15 (0.80, 1.56)	1.09 (0.67, 1.48)	0.629
HDL (mmol/L), median (Q1, Q3)	1.08 (0.95, 1.30)	1.10 (0.92, 1.22)	0.598
LDL (mmol/L), median (Q1, Q3)	2.70 (2.24, 3.19)	2.37 (2.00, 2.71)	0.056
ALB (g/L), mean ± SD	37.3 ± 4.0	35.5 ± 4.4	0.149
UREA (mmol/L), median (Q1, Q3)	6.5 (5.1, 7.6)	8.8 (5.7, 14.9)	0.050
CREA (μmol/L), median (Q1, Q3)	76 (63, 105)	114 (67, 284)	0.064
RRR, median (Q1, Q3)	2.84 (2.63, 3.17)	3.51 (3.04, 3.90)	< 0.001

*Note:* ALB: albumin; ALP: alkaline phosphatase; ALT: alanine aminotransferase; AST: aspartate aminotransferase; CREA: creatinine; DBIL: direct bilirubin; HGB: hemoglobin; LDH: lactate dehydrogenase; LY: lymphocytes; MO: monocytes; NE: neutrophils; PLT: platelets; RDW: red cell distribution width; RRR: red cell distribution width to red blood cell ratio; TBIL: total bilirubin; TG: triglycerides; UREA: urea nitrogen; WBC: leukocyte.

Abbreviations: CK, creatine kinase; HDL, high‐density lipoprotein; LDL, low‐density lipoprotein; RBC, red blood cell; TC, total cholesterol.

### 3.2. Feature Selection and Multivariate Analysis

**FIGURE 1:**
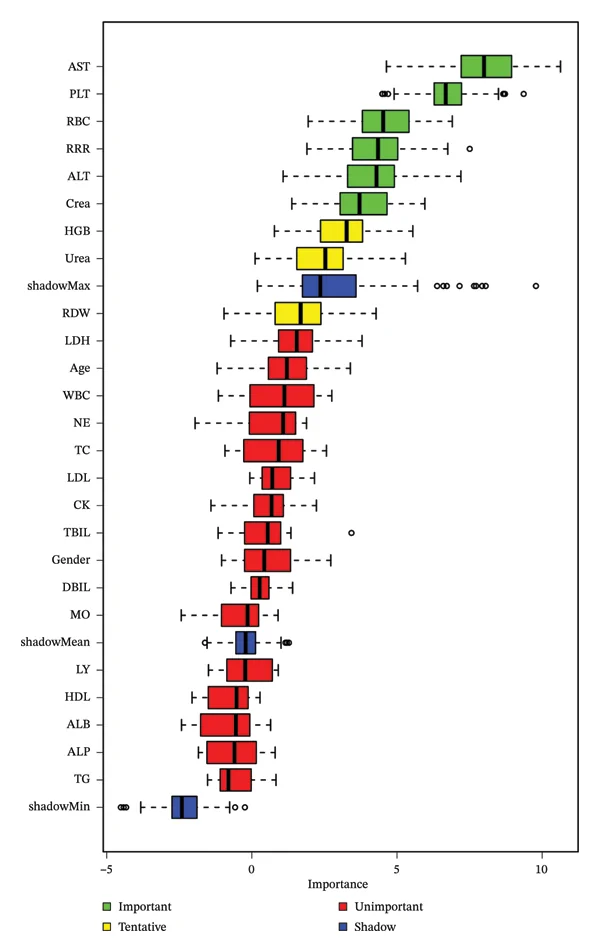
Feature selected with Boruta. Boruta algorithm identified six predictors: RRR, AST, RBC, PLT, ALT, and CREA.

The Boruta algorithm identified six predictors as “confirmed” important features: RRR, AST, RBC, PLT, ALT, and CREA (Figure 1). Subsequent VIF analysis revealed that RBC (VIF = 5.4) and AST (VIF = 12.4) exceeded the threshold of 5, indicating severe multicollinearity. Consequently, RBC and AST were excluded from the final model. The remaining four variables—RRR, ALT, PLT, and CREA—were entered into a multivariable logistic regression model. After adjustment for these confounding factors, RRR remained the only variable independently associated with 30‐day mortality (odds ratio [OR] = 3.059; 95% CI: 1.431–6.540; *p* = 0.004) (Table 2).

**TABLE 2:** Multivariate logistic regression of 30‐day mortality for patients with BAAD.

Variables	Multivariate analysis		
	OR	95% CI	*p* value
RRR	3.059	1.431–6.540	0.004
PLT	0.993	0.983–1.004	0.189
ALT	1.000	0.998–1.002	0.826
CREA	1.001	0.994–1.008	0.859

*Note.* ALT: alanine aminotransferase; AUC: area under the curve; CREA: creatinine; PLT: platelets; RRR: red cell distribution width to red blood cell ratio.

Abbreviations: CI, confidence interval; OR, odds ratio.

### 3.3. ROC Analysis

**FIGURE 2:**
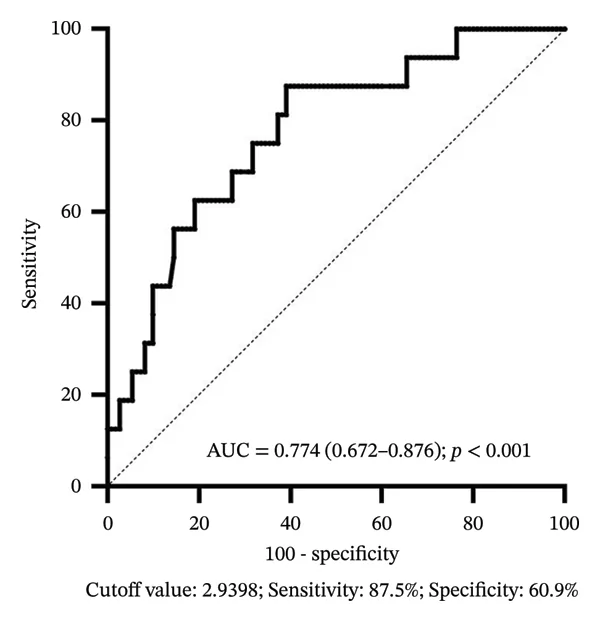
ROC curve for RRR in predicting 30‐day mortality.

For predicting 30‐day mortality, the area under the curve (AUC) for RRR was 0.774 (95% confidence interval [CI]: 0.672–0.876; *p* < 0.001), indicating good predictive discrimination (Figure 1). The exploratory threshold of 2.94 yielded a sensitivity of 87.5% and a specificity of 60.9%. This cutoff value is derived from our specific cohort and requires validation in independent populations before any clinical application. However, the prognostic performance of RRR, as measured by its AUC (0.774), was not statistically superior to that of RDW alone (AUC = 0.684; DeLong’s test, *p* = 0.155) and RBC alone (AUC = 0.741, DeLong’s test, *p* = 0.335) (Figure 2).

## 4. Discussion

This study represents the first systematic investigation into the prognostic utility of RRR specifically in patients with BAAD. Our analysis revealed that patients with an elevated RRR had a markedly higher 30‐day mortality rate compared to those with a lower ratio. Furthermore, after rigorous feature selection using the Boruta algorithm and exclusion of collinear variables via VIF analysis, multivariate analysis confirmed that RRR was independently associated with short‐term mortality within our laboratory‐based model. However, given the preliminary nature of these findings, they should be interpreted as hypothesis‐generating rather than definitive.

An elevated RDW is a recognized marker of systemic inflammation, oxidative stress, impaired erythropoiesis, and nutritional deficits [6]. Prior research has identified RDW as an independent prognostic indicator for all‐cause and cardiovascular mortality across various conditions, including breast cancer, nonalcoholic fatty liver disease, and sepsis [9–11]. Within the context of aortic disease, inflammatory cytokines are known to inflict direct endothelial injury, thereby increasing aortic wall vulnerability. Concurrently, these cytokines can disrupt erythropoietin signaling and iron homeostasis, leading to anisocytosis and elevated RDW [12–14]. Our findings are consistent with this established pathophysiological link.

As the principal cellular component of blood, erythrocytes are essential for oxygen transport. A diminished RBC count, indicative of anemia, directly compromises the blood’s oxygen‐carrying capacity. In the acute phase of BAAD, where organ malperfusion frequently occurs due to dynamic true lumen compression, concomitant anemia may exacerbate tissue ischemia and hypoxia, potentially establishing a detrimental cycle.

The novelty of our work lies in integrating RDW and RBC counts into a single composite ratio and applying it specifically to the BAAD prognosis. The RRR demonstrated good discriminative ability for 30‐day mortality, with an AUC of 0.774. Its prognostic value likely arises from its concurrent assessment of two key pathophysiological dimensions: the systemic inflammatory/stress state (reflected by RDW) and the circulatory oxygen‐carrying potential (reflected by RBC count). An elevated RRR may thus signify a high‐risk confluence of heightened inflammatory dysregulation and relative anemia—a state particularly deleterious in the stress of an acute aortic syndrome.

To identify robust predictors in this exploratory analysis and mitigate the risk of overfitting associated with our modest sample size, we employed a two‐step statistical approach. First, the Boruta algorithm was used for feature selection by comparing the importance of actual variables against randomly permuted “shadow” features. From a comprehensive panel of laboratory parameters, Boruta identified six key variables, including RRR. Second, VIF analysis was performed to detect multicollinearity among the selected variables. As expected, AST and RBC showed VIF values exceeding 5 and were, therefore, excluded to ensure model stability. In final multivariate logistic regression, RRR retained an independent and statistically significant association with 30‐day mortality within our laboratory‐based model. However, we acknowledge that because variable selection and model estimation were performed in the same cohort without internal or external validation, the risk of overfitting cannot be entirely excluded. Our findings should, therefore, be considered hypothesis‐generating, providing a foundation for future investigations.

It is noteworthy that traditional predictors such as age and HGB were not retained in the final model after the Boruta and VIF selection process. This likely reflects the prioritization of variables with the strongest predictive signal by the Boruta algorithm in our specific study, as RRR emerged as the most robust among correlated hematological parameters. However, this exclusion could also be influenced by our modest sample size, the limited number of outcome events, and the inherent instability of data‐driven variable selection procedures. Therefore, this finding does not imply that age or HGB are clinically irrelevant. Furthermore, we did not perform a formal mediation analysis, and any suggestion that RRR mediates the prognostic information of its individual components would be speculative and unsupported by our study design.

The potential clinical implications of this finding merit consideration but must be interpreted with caution. Calculated from standard complete blood count parameters, RRR is a simple, rapid, and inexpensive metric universally available upon hospital admission. However, it is important to emphasize that our findings are hypothesis‐generating rather than definitive. The RRR should currently be considered a candidate prognostic marker requiring prospective validation before clinical implementation. If validated in future studies, RRR might one day function as a bedside risk stratification tool for BAAD patients. However, at present, its clinical utility remains unproven, and any application to clinical decision‐making would be premature.

Our findings demonstrate an association between RRR and mortality rather than established clinical prediction. The modest AUC of 0.774 suggests that RRR alone is insufficient as a standalone prediction tool. Furthermore, RRR is a nonspecific biomarker influenced by various conditions beyond aortic dissection. Its incremental value beyond established clinical risk scores requires formal evaluation in future prospective studies.

Several limitations warrant consideration. This was a single‐center retrospective study with a modest sample size (*n* = 126) and only 16 outcome events, limiting statistical power, generalizability and introducing possible selection bias. Our four‐variable model (RRR, ALT, PLT, and CREA) yielded an events‐per‐variable ratio of 4:1, below the ideal threshold. We also lacked adjustment for critical clinical confounders, including dissection complexity, malperfusion, rupture, aortic diameter, hemodynamic status, treatment strategy, and major comorbidities. Moreover, only baseline RRR was assessed without tracking its dynamic changes. These findings are hypothesis‐generating and require validation in larger, multicenter, prospective cohorts.

## 5. Conclusion

In summary, our study demonstrates that the RRR is associated with 30‐day mortality in patients with BAAD and might represent a candidate prognostic marker. Readily derived from routine blood tests, it provides a cost‐effective reflection of an integrated pathophysiology involving inflammation and oxygen transport capacity. However, given the preliminary nature of this single‐center retrospective analysis with a modest sample size, clinical application of RRR as a risk stratification tool would be premature. Prospective validation in larger, multicenter cohorts is urgently needed before this biomarker can be recommended for clinical decision‐making. At present, our findings should be considered hypothesis‐generating, providing a foundation for future investigations.

## Author Contributions

Jun Zhou designed the research study. Jun Zhou performed the research. Jun Zhou and Jingjing Wang analyzed the data. Jingjing Wang wrote the manuscript. All authors contributed to editorial changes in the manuscript.

## Funding

No funding was received for this manuscript.

## Disclosure

All authors read and approved the final manuscript.

## Ethics Statement

The study was performed to conform with the Declaration of Helsinki and was approved by the local ethics committee of the hospital (2021‐SR‐529).

## Conflicts of Interest

The authors declare no conflicts of interest.

## Supporting Information

Additional supporting information can be found online in the Supporting Information section.

## Supporting information


**Supporting Information** STROBE_checklist_cohort.

## Data Availability

The anonymized data that support the findings of this study are available from the corresponding author upon reasonable request, subject to institutional ethical approval.

## References

[1] Wang M. M. , Gai M. T. , Wang B. Z. , Yesitayi G. , Ma Y. T. , and Ma X. , A Prediction Model to Predict in-Hospital Mortality in Patients With Acute Type B Aortic Dissection, BMC Cardiovascular Disorders. (2023) 23, no. 1, 10.1186/s12872-023-03260-5. PMC10193648 37198546

[2] Cooper M. , Hicks C. , Ratchford E. V. , Salameh M. J. , and Malas M. , Diagnosis and Treatment of Uncomplicated Type B Aortic Dissection, Vascular Medicine. (2016) 21, no. 6, 547–552, 10.1177/1358863x16643601. 27126951

[3] Tang X. , Qiu S. , Sun X. , Yang G. , and Sheng L. , The Prognostic Impact of Maximal Aortic Diameter on Acute Type B Aortic Dissection Progression in a Chinese Population, Scientific Reports. (2024) 14, no. 1, 10.1038/s41598-024-77649-3. PMC11513997 39462034

[4] Guray Y. , Ipek E. G. , Guray U. et al., Red Cell Distribution Width Predicts Mortality in Infective Endocarditis, Arch Cardiovasc Dis. (2014) 107, no. 5, 299–307, 10.1016/j.acvd.2014.04.008. 24923758

[5] Xie K. H. , Liu L. L. , Liang Y. R. et al., Red Cell Distribution Width: A Novel Predictive Biomarker for Stroke Risk After Transient Ischaemic Attack, Annals of Medicine. (2022) 54, no. 1, 1167–1177, 10.1080/07853890.2022.2059558. PMC9045760 35471128

[6] Garcia-Escobar A. , Lazaro-Garcia R. , Goicolea-Ruigomez J. et al., Red Blood Cell Distribution Width is a Biomarker of Red Cell Dysfunction Associated With High Systemic Inflammation and a Prognostic Marker in Heart Failure and Cardiovascular Disease: A Potential Predictor of Atrial Fibrillation Recurrence, High Blood Pressure and Cardiovascular Prevention. (2024) 31, no. 5, 437–449, 10.1007/s40292-024-00662-0. 39031283

[7] Xu W. , Huo J. , Chen G. et al., Association Between Red Blood Cell Distribution Width to Albumin Ratio and Prognosis of Patients With Sepsis: A Retrospective Cohort Study, Frontiers in Nutrition. (2022) 9, 10.3389/fnut.2022.1019502. PMC9539557 36211519

[8] Scalco R. , de Oliveira G. N. , da Rosa C. B. et al., Red Blood Cell Distribution Width to Platelet Ratio in Neonatal Foals With Sepsis, Journal of Veterinary Internal Medicine. (2023) 37, no. 4, 1552–1560, 10.1111/jvim.16793. PMC10365058 37306395

[9] Liu X. , Wu H. , Zhang S. , Zhu Z. , Liu C. , and Cao J. , Higher Red Cell Distribution Width (RDW) is Associated with Increased All-Cause and Cardiovascular Mortality in Patients With Breast Cancer: A Retrospective Analysis of NHANES Data (1999-2018), PLoS One. (2025) 20, no. 7, 10.1371/journal.pone.0328680. PMC12303287 40720508

[10] Huang Y. , Ao T. , Wang Y. , Zhen P. , and Hu M. , The Red Blood Cell Distribution Width is Associated With All-Cause and Cardiovascular Mortality Among Individuals With Non-Alcoholic Fatty Liver Disease, PLoS One. (2025) 20, no. 4, 10.1371/journal.pone.0321789. PMC12005546 40245046

[11] Zhang L. , Yu C. H. , Guo K. P. , Huang Cz , and Mo Ly , Prognostic Role of Red Blood Cell Distribution Width in Patients With Sepsis: A Systematic Review and Meta-Analysis, BMC Immunology. (2020) 21, no. 1, 10.1186/s12865-020-00369-6. PMC7339553 32631218

[12] Sano M. and Anzai J. , [The Molecular Mechanisms Contributing to the Pathophysiology of Systemic Inflammatory Response After Acute Aortic Dissection], Nihon Rinsho Meneki Gakkai Kaishi. (2016) 39, no. 2, 91–95, 10.2177/jsci.39.91. 27212594

[13] Saad H. , Soliman H. A. , Mahmoud B. , Moneim A. A. , and Zaky M. Y. , The Pathogenic Role of Oxidative Stress, Cytokine Expression, and Impaired Hematological Indices in Diabetic Cardiovascular Diseases, Inflammation. (2023) 46, no. 1, 146–160, 10.1007/s10753-022-01718-w. PMC9971070 35997998

[14] Rhodes C. J. , Wharton J. , and Wilkins M. R. , Pulmonary Hypertension: Biomarkers, Handbook of Experimental Pharmacology. (2013) 218, 77–103. 10.1007/978-3-642-38664-0_4 24092337

